# Supplementary material for: The Effect of Early Life Stress on Emotional Behaviors in GPR37KO Mice
Source: Int J Mol Sci. 2021 Dec 30;23(1):410. doi: 10.3390/ijms23010410 (PMC8745300; doi:10.3390/ijms23010410)
Supplement: Supplementary file 1 [file ijms-23-00410-s001.zip › ijms-1496877-supplementary.pdf]

**Supplementary Materials**

**Raw statistics of the data discussed in the manuscript.**

**Table S1.** Number of visits in open arms in EPM.

| ANOVA table | F (DFn, DFd) | P value |
|---|---|---|
| sex | F (1, 57) = 1.064 | P=0.3066 |
| genotype | F (1, 57) = 0.02513 | P=0.8746 |
| treatment | F (1, 57) = 4.780 | P=0.0329 |
| sex x genotype | F (1, 57) = 1.504 | P=0.2252 |
| sex x treatment | F (1, 57) = 0.1896 | P=0.6649 |
| genotype x treatment | F (1, 57) = 2.642 | P=0.1096 |
| sex x genotype x treatment | F (1, 57) = 0.3658 | P=0.5477 |

| | |
|---|---|
| Two-stage linear step-up procedure of Benjamini, Krieger and Yekutieli | Individual P Value |
| Male:Freq in open arms WT no ELS vs. Male:Freq in open arms WT ELS | 0.2021 |
| Male:Freq in open arms WT no ELS vs. Male:Freq in open arms KO no ELS | 0.2632 |
| Male:Freq in open arms WT no ELS vs. Female:Freq in open arms WT no ELS | 0.672 |
| Male:Freq in open arms WT ELS vs. Male:Freq in open arms KO ELS | 0.982 |
| Male:Freq in open arms WT ELS vs. Female:Freq in open arms WT ELS | 0.579 |
| Male:Freq in open arms KO no ELS vs. Male:Freq in open arms KO ELS | 0.694 |
| Male:Freq in open arms KO no ELS vs. Female:Freq in open arms KO no ELS | 0.263 |
| Male:Freq in open arms KO ELS vs. Female:Freq in open arms KO ELS | 0.214 |
| Female:Freq in open arms WT no ELS vs. Female:Freq in open arms WT ELS | 0.0219 |
| Female:Freq in open arms WT no ELS vs. Female:Freq in open arms KO no ELS | 0.672 |
| Female:Freq in open arms WT ELS vs. Female:Freq in open arms KO ELS | 0.076 |
| Female:Freq in open arms KO no ELS vs. Female:Freq in open arms KO ELS | 0.840 |

**Table S2.** Time in open arms (%) in EPM.

| ANOVA table | F (DFn, DFd) | P value |
|---|---|---|
| sex | F (1, 56) = 0.9210 | P=0.3413 |
| genotype | F (1, 56) = 0.2590 | P=0.6128 |
| treatment | F (1, 56) = 0.6651 | P=0.4182 |
| sex x genotype | F (1, 56) = 0.2952 | P=0.5890 |
| sex x treatment | F (1, 56) = 0.04045 | P=0.8413 |
| genotype x treatment | F (1, 56) = 3.137 | P=0.0820 |
| sex x genotype x treatment | F (1, 56) = 4.871 | P=0.0314 |

| Two-stage linear step-up procedure of Benjamini, Krieger and Yekutieli | Individual P Value |
|---|---|
| Male:WT no ELS vs. Male:WT ELS | 0.7860 |
| Male:WT no ELS vs. Male:KO no ELS | 0.8274 |
| Male:WT no ELS vs. Female:WT no ELS | 0.2243 |
| Male:WT ELS vs. Male:KO ELS | 0.8260 |
| Male:WT ELS vs. Female:WT ELS | 0.4717 |
| Male:KO no ELS vs. Male:KO ELS | 0.4312 |
| Male:KO no ELS vs. Female:KO no ELS | 0.6368 |
| Male:KO ELS vs. Female:KO ELS | 0.0459 |
| Female:WT no ELS vs. Female:WT ELS | 0.0283 |
| Female:WT no ELS vs. Female:KO no ELS | 0.1571 |
| Female:WT ELS vs. Female:KO ELS | 0.0142 |
| Female:KO no ELS vs. Female:KO ELS | 0.0960 |

**Table S3.** Number of visits in light dark box.

| ANOVA table | F (DFn, DFd) | P value |
|---|---|---|
| sex | F (1, 59) = 1.397 | P=0.2419 |
| genotype | F (1, 59) = 3.383 | P=0.0709 |
| treatment | F (1, 59) = 0.1641 | P=0.6868 |
| sex x genotype | F (1, 59) = 12.29 | P=0.0009 |
| sex x treatment | F (1, 59) = 0.1377 | P=0.7119 |
| genotype x treatment | F (1, 59) = 2.461 | P=0.1220 |
| sex x genotype x treatment | F (1, 59) = 0.4959 | P=0.4841 |

| Two-stage linear step-up procedure of Benjamini, Krieger and Yekutieli | Individual P Value |
|---|---|
| Male:WT no ELS vs. Male:WT ELS | 0.274 |
| Male:WT no ELS vs. Male:KO no ELS | 0.044 |
| Male:WT no ELS vs. Female:WT no ELS | 0.015 |
| Male:WT ELS vs. Male:KO ELS | 0.761 |
| Male:WT ELS vs. Female:WT ELS | 0.057 |
| Male:KO no ELS vs. Male:KO ELS | 0.219 |
| Male:KO no ELS vs. Female:KO no ELS | 0.072 |
| Male:KO ELS vs. Female:KO ELS | 0.503 |
| Female:WT no ELS vs. Female:WT ELS | 0.454 |
| Female:WT no ELS vs. Female:KO no ELS | 0.0260 |
| Female:WT ELS vs. Female:KO ELS | 0.005 |
| Female:KO no ELS vs. Female:KO ELS | 0.963 |

**Table S4.** Time in light chamber (%) in light dark box.

| ANOVA table | F (DFn, DFd) | P value |
|---|---|---|
| sex | F (1, 57) = 8.165 | P=0.0060 |
| genotype | F (1, 57) = 1.780 | P=0.1874 |
| treatment | F (1, 57) = 2.189 | P=0.1445 |
| sex x genotype | F (1, 57) = 0.1840 | P=0.6695 |
| sex x treatment | F (1, 57) = 0.8417 | P=0.3628 |
| genotype x treatment | F (1, 57) = 8.670 | P=0.0047 |
| sex x genotype x treatment | F (1, 57) = 1.326 | P=0.2543 |

| Two-stage linear step-up procedure of Benjamini, Krieger and Yekutieli | Individual P Value |
|---|---|
| Male:WT no ELS vs. Male:WT ELS | 0.2713 |
| Male:WT no ELS vs. Male:KO no ELS | 0.628 |
| Male:WT no ELS vs. Female:WT no ELS | 0.568 |
| Male:WT ELS vs. Male:KO ELS | 0.188 |
| Male:WT ELS vs. Female:WT ELS | 0.021 |
| Male:KO no ELS vs. Male:KO ELS | 0.475 |
| Male:KO no ELS vs. Female:KO no ELS | 0.136 |
| Male:KO ELS vs. Female:KO ELS | 0.228 |
| Female:WT no ELS vs. Female:WT ELS | 0.005 |
| Female:WT no ELS vs. Female:KO no ELS | 0.264 |
| Female:WT ELS vs. Female:KO ELS | 0.006 |
| Female:KO no ELS vs. Female:KO ELS | 0.362 |

**Table S5.** Training latency (sec) in Passive avoidance task.

| ANOVA table | F (DFn, DFd) | P value |
|---|---|---|
| sex | F (1, 61) = 12.33 | P=0.0008 |
| genotype | F (1, 61) = 1.424 | P=0.2373 |
| treatment | F (1, 61) = 1.302 | P=0.2583 |
| sex x genotype | F (1, 61) = 1.262 | P=0.2656 |
| sex x treatment | F (1, 61) = 0.2376 | P=0.6277 |
| genotype x treatment | F (1, 61) = 0.6645 | P=0.4181 |
| sex x genotype x treatment | F (1, 61) = 0.07811 | P=0.7808 |

| Two-stage linear step-up procedure of Benjamini, Krieger and Yekutieli | Individual P Value |
|---|---|
| Male:WT no ELS vs. Male:WT ELS | 0.8319 |
| Male:WT no ELS vs. Male:KO no ELS | 0.5103 |
| Male:WT no ELS vs. Female:WT no ELS | 0.1191 |
| Male:WT ELS vs. Male:KO ELS | 0.0969 |
| Male:WT ELS vs. Female:WT ELS | 0.4632 |
| Male:KO no ELS vs. Male:KO ELS | 0.3337 |
| Male:KO no ELS vs. Female:KO no ELS | 0.0137 |
| Male:KO ELS vs. Female:KO ELS | 0.0222 |
| Female:WT NO ELS vs. Female:WT ELS | 0.6090 |
| Female:WT NO ELS vs. Female:KO NO ELS | 0.8213 |
| Female:WT ELS vs. Female:KO ELS | 0.7708 |
| Female:KO NO ELS vs. Female:KO ELS | 0.2824 |

**Table S6. Test latency (sec) in Passive avoidance task.**

| ANOVA table | F (DFn, DFd) | P value |
|---|---|---|
| sex | F (1, 60) = 2.865 | P=0.0957 |
| genotype | F (1, 60) = 6.588 | P=0.0128 |
| treatment | F (1, 60) = 3.242 | P=0.0768 |
| sex x genotype | F (1, 60) = 0.4559 | P=0.5021 |
| sex x treatment | F (1, 60) = 2.713 | P=0.1047 |
| genotype x treatment | F (1, 60) = 0.02093 | P=0.8854 |
| sex x genotype x treatment | F (1, 60) = 1.215 | P=0.2748 |

| Two-stage linear step-up procedure of Benjamini, Krieger and Yekutieli | Individual P Value |
|---|---|
| Male:WT NO ELS vs. Male:WT ELS | 0.0368 |
| Male:WT NO ELS vs. Male:KO NO ELS | 0.2411 |
| Male:WT NO ELS vs. Female:WT NO ELS | 0.0630 |
| Male:WT ELS vs. Male:KO ELS | 0.0416 |
| Male:KO no ELS vs. Male:KO ELS | 0.190 |
| Male:WT ELS vs. Female:WT ELS | 0.4317 |
| Male:KO NO ELS vs. Female:KO NO ELS | 0.1363 |
| Male:KO ELS vs. Female:KO ELS | 0.3363 |
| Female:WT NO ELS vs. Female:WT ELS | 0.6076 |
| Female:WT NO ELS vs. Female:KO NO ELS | 0.1198 |
| Female:WT ELS vs. Female:KO ELS | 0.7557 |
| Female:KO NO ELS vs. Female:KO ELS | 0.4708 |

**Table S7. Time spent immobile (%) in FST.**

| ANOVA table | F (DFn, DFd) | P value |
|---|---|---|
| sex | F (1, 57) = 1.830 | P=0.1815 |
| genotype | F (1, 57) = 5.633 | P=0.0210 |
| treatment | F (1, 57) = 1.842 | P=0.1801 |
| sex x genotype | F (1, 57) = 0.09588 | P=0.7580 |
| sex x treatment | F (1, 57) = 3.108 | P=0.0833 |
| genotype x treatment | F (1, 57) = 2.611 | P=0.1116 |
| sex x genotype x treatment | F (1, 57) = 2.289 | P=0.1358 |

| Two-stage linear step-up procedure of Benjamini, Krieger and Yekutieli | Individual P Value |
|---|---:|
| Male:WT NO ELS vs. Male:WT ELS | 0.0941 |
| Male:WT NO ELS vs. Male:KO NO ELS | 0.0100 |
| Male:WT NO ELS vs. Female:WT NO ELS | 0.0356 |
| Male:WT ELS vs. Male:KO ELS | 0.6004 |
| Male:WT ELS vs. Female:WT ELS | 0.3022 |
| Male:KO NO ELS vs. Male:KO ELS | 0.1554 |
| Male:KO NO ELS vs. Female:KO NO ELS | 0.3308 |
| Male:KO ELS vs. Female:KO ELS | 0.4583 |
| Female:WT NO ELS vs. Female:WT ELS | 0.1517 |
| Female:WT NO ELS vs. Female:KO NO ELS | 0.170 |
| Female:WT ELS vs. Female:KO ELS | 0.2071 |
| Female:KO NO ELS vs. Female:KO ELS | 0.1038 |

**Table S8. Body weight (P12).**

| ANOVA table | F (DFn, DFd) | P value |
|---|---|---|
| interaction | F (1, 58) = 0.07953 | P=0.7789 |
| treatment | F (1, 58) = 14.30 | P=0.0004 |
| group | F (1, 58) = 4.546 | P=0.0372 |

| Two-stage linear step-up procedure of Benjamini, Krieger and Yekutieli | Individual P Value |
|---|---:|
| NO ELS:WT vs. NO ELS:GPR37KO | 0.1617 |
| NO ELS:WT vs. ELS:WT | 0.0082 |
| NO ELS:WT vs. ELS:GPR37KO | <0.0001 |
| NO ELS:GPR37KO vs. ELS:WT | 0.2496 |
| NO ELS:GPR37KO vs. ELS:GPR37KO | 0.0106 |
| ELS:WT vs. ELS:GPR37KO | 0.1166 |

**Table S9. Body weight (adulthood).**

| ANOVA table | F (DFn, DFd) | P value |
|---|---|---|
| sex | F (1, 67) = 142.7 | P<0.0001 |
| genotype | F (1, 67) = 0.001435 | P=0.9699 |
| treatment | F (1, 67) = 8.612 | P=0.0046 |
| sex x genotype | F (1, 67) = 6.489 | P=0.0132 |
| sex x treatment | F (1, 67) = 0.1079 | P=0.7436 |
| genotype x treatment | F (1, 67) = 0.3398 | P=0.5619 |
| sex x genotype x treatment | F (1, 67) = 1.128 | P=0.2921 |

| Two-stage linear step-up procedure of Benjamini, Krieger and Yekutieli | Individual P Value |
|---|---|
| Male:WT NO ELS vs. Male:WT ELS | 0.1478 |
| Male:WT NO ELS vs. Male:KO NO ELS | 0.2895 |
| Male:WT NO ELS vs. Female:WT NO ELS | <0.0001 |
| Male:WT ELS vs. Male:KO ELS | 0.1585 |
| Male:WT ELS vs. Female:WT ELS | <0.0001 |
| Male:KO NO ELS vs. Male:KO ELS | 0.0786 |
| Male:KO NO ELS vs. Female:KO NO ELS | <0.0001 |
| Male:KO ELS vs. Female:KO ELS | 0.0005 |
| Female:WT NO ELS vs. Female:WT ELS | 0.0425 |
| Female:WT NO ELS vs. Female:KO NO ELS | 0.5980 |
| Female:WT ELS vs. Female:KO ELS | 0.0588 |
| Female:KO NO ELS vs. Female:KO ELS | 0.6220 |

**Table S10. P-T286-CaMKII/total CaMKII in the DH.**

| ANOVA table | F (DFn, DFd) | P value |
|---|---|---|
| TREATMENT | F (2, 64) = 0.5735 | P=0.5664 |
| SEX | F (1, 64) = 3.619 | P=0.0616 |
| GENOTYPE | F (1, 64) = 1.809e-005 | P=0.9966 |
| TREATMENT x SEX | F (2, 64) = 2.235 | P=0.1153 |
| TREATMENT x GENOTYPE | F (2, 64) = 2.542 | P=0.0866 |
| SEX x GENOTYPE | F (1, 64) = 1.772 | P=0.1879 |
| TREATMENT x SEX x GENOTYPE | F (2, 64) = 0.03411 | P=0.9665 |

| Two-stage linear step-up procedure of Benjamini, Krieger and Yekutieli | Individual P Value |
|---|---|
| Basal:Male WT vs. Basal:Male KO | 0.1666 |
| Basal:Male WT vs. Basal:Female WT | >0.9999 |
| Basal:Male WT vs. No ELS:Male WT | 0.2689 |
| Basal:Male WT vs. ELS:Male WT | 0.8208 |
| Basal:Male KO vs. Basal:Female KO | 0.4284 |
| Basal:Male KO vs. No ELS:Male KO | 0.1344 |
| Basal:Male KO vs. ELS:Male KO | 0.0293 |
| Basal:Male KO vs. ELS:Female KO | 0.2450 |
| Basal:Female WT vs. Basal:Female KO | 0.5998 |
| Basal:Female WT vs. No ELS:Female WT | 0.5493 |
| Basal:Female WT vs. ELS:Female WT | 0.0486 |
| Basal:Female KO vs. No ELS:Female KO | 0.8402 |
| Basal:Female KO vs. ELS:Female KO | 0.7181 |
| No ELS:Male WT vs. No ELS:Male KO | 0.3218 |
| No ELS:Male WT vs. No ELS:Female WT | 0.0947 |
| No ELS:Male WT vs. ELS:Male WT | 0.4018 |
| No ELS:Male KO vs. No ELS:Female KO | 0.6547 |
| No ELS:Male KO vs. ELS:Male KO | 0.4422 |
| No ELS:Female WT vs. ELS:Female WT | 0.1637 |
| No ELS:Female KO vs. ELS:Female KO | 0.8733 |
| ELS:Male WT vs. ELS:Male KO | 0.5283 |
| ELS:Male WT vs. ELS:Female WT | 0.0293 |
| ELS:Male KO vs. ELS:Female KO | 0.3125 |
| ELS:Female WT vs. ELS:Female KO | 0.0695 |

**Table S11. P-T286-CaMKII/total CaMKII in the VH.**

| ANOVA table | F (DFn, DFd) | P value |
|---|---|---|
| TREATMENT | F (2, 61) = 2.262 | P=0.1129 |
| SEX | F (1, 61) = 0.05368 | P=0.8176 |
| GENOTYPE | F (1, 61) = 2.580 | P=0.1134 |
| TREATMENT x SEX | F (2, 61) = 3.193 | P=0.0480 |
| TREATMENT x GENOTYPE | F (2, 61) = 1.618 | P=0.2068 |
| SEX x GENOTYPE | F (1, 61) = 0.09911 | P=0.7540 |
| TREATMENT x SEX x GENOTYPE | F (2, 61) = 3.109 | P=0.0518 |

| Two-stage linear step-up procedure of Benjamini, Krieger and Yekutieli | Individual P Value |
|---|---|
| Basal:Male WT vs. Basal:Male KO | 0.0013 |
| Basal:Male WT vs. Basal:Female WT | >0.9999 |
| Basal:Male WT vs. No ELS:Male WT | 0.0823 |
| Basal:Male WT vs. ELS:Male WT | 0.1130 |
| Basal:Male KO vs. Basal:Female KO | 0.0034 |
| Basal:Male KO vs. No ELS:Male KO | 0.0241 |
| Basal:Male KO vs. ELS:Male KO | 0.0507 |
| Basal:Female WT vs. Basal:Female KO | 0.8853 |
| Basal:Female WT vs. No ELS:Female WT | 0.1850 |
| Basal:Female WT vs. ELS:Female WT | 0.0528 |
| Basal:Female KO vs. No ELS:Female KO | 0.0452 |
| Basal:Female KO vs. ELS:Male KO | 0.2698 |
| Basal:Female KO vs. ELS:Female KO | 0.0261 |
| No ELS:Male WT vs. No ELS:Male KO | 0.5176 |
| No ELS:Male WT vs. No ELS:Female WT | 0.7088 |
| No ELS:Male WT vs. ELS:Male WT | 0.9225 |
| No ELS:Male KO vs. No ELS:Female KO | 0.2189 |
| No ELS:Male KO vs. ELS:Male KO | 0.8200 |
| No ELS:Female WT vs. No ELS:Female KO | 0.3429 |
| No ELS:Female WT vs. ELS:Female WT | 0.4886 |
| No ELS:Female KO vs. ELS:Female KO | 0.8061 |
| ELS:Male WT vs. ELS:Female WT | 0.6603 |
| ELS:Male KO vs. ELS:Female KO | 0.2259 |
| ELS:Female WT vs. ELS:Female KO | 0.6544 |

**Table S12. P-T286-CaMKII/total CaMKII in the amygdala.**

| ANOVA table | F (DFn, DFd) | P value |
|---|---|---|
| TREATMENT | F (2, 64) = 5.977 | P=0.0042 |
| SEX | F (1, 64) = 0.7630 | P=0.3856 |
| GENOTYPE | F (1, 64) = 0.01879 | P=0.8914 |
| TREATMENT x SEX | F (2, 64) = 0.7687 | P=0.4679 |
| TREATMENT x GENOTYPE | F (2, 64) = 0.4362 | P=0.6484 |
| SEX x GENOTYPE | F (1, 64) = 2.635 | P=0.1095 |
| TREATMENT x SEX x GENOTYPE | F (2, 64) = 3.020 | P=0.0558 |

| Two-stage linear step-up procedure of Benjamini, Krieger and Yekutieli | Individual P Value |
|---|---:|
| Basal:Male WT vs. Basal:Male KO | 0.4506 |
| Basal:Male WT vs. Basal:Female WT | >0.9999 |
| Basal:Male WT vs. No ELS:Male WT | 0.0350 |
| Basal:Male WT vs. No ELS:Male KO | 0.4351 |
| Basal:Male WT vs. ELS:Male WT | 0.2016 |
| Basal:Male KO vs. Basal:Female KO | 0.2244 |
| Basal:Male KO vs. No ELS:Male KO | 0.9555 |
| Basal:Male KO vs. ELS:Male KO | 0.4696 |
| Basal:Male KO vs. ELS:Female KO | 0.5026 |
| Basal:Female WT vs. Basal:Female KO | 0.6415 |
| Basal:Female WT vs. No ELS:Female WT | 0.4953 |
| Basal:Female WT vs. ELS:Female WT | 0.7347 |
| Basal:Female KO vs. No ELS:Female KO | 0.0009 |
| Basal:Female KO vs. ELS:Female KO | 0.6362 |
| No ELS:Male WT vs. No ELS:Male KO | 0.1933 |
| No ELS:Male WT vs. No ELS:Female WT | 0.1286 |
| No ELS:Male WT vs. ELS:Male WT | 0.4412 |
| No ELS:Male KO vs. No ELS:Female KO | 0.0334 |
| No ELS:Male KO vs. ELS:Male KO | 0.4530 |
| No ELS:Female WT vs. No ELS:Female KO | 0.0176 |
| No ELS:Female WT vs. ELS:Female WT | 0.3375 |
| No ELS:Female KO vs. ELS:Female KO | 0.0066 |
| ELS:Male WT vs. ELS:Male KO | 0.2208 |
| ELS:Male WT vs. ELS:Female WT | 0.1238 |
| ELS:Male KO vs. ELS:Female KO | 0.9608 |
| ELS:Female WT vs. ELS:Female KO | 0.7211 |
